# CXCL5: A coachman to drive cancer progression

**DOI:** 10.3389/fonc.2022.944494

**Published:** 2022-08-01

**Authors:** Jie Deng, Rongqi Jiang, Enqing Meng, Hao Wu

**Affiliations:** Department of Oncology, The First Affiliated Hospital of Nanjing Medical University, Nanjing, China

**Keywords:** CXCL5, chemokine, immunosuppressive microenvironment, tumor angiogenesis, tumor migration

## Abstract

Chemokines are a class of pro-inflammatory cytokines that can recruit and activate chemotactic cells. C‐X‐C motif chemokine ligand 5 (CXCL5) is a member of the chemokine family binding CXCR2 (C-X-C Motif Chemokine Receptor 2), a G-protein coupled receptor. Accumulated evidence has shown that dysregulated CXCL5 participates in tumor metastasis and angiogenesis in human malignant tumors. In this review, we summarized the advances in research on CXCL5, including its dysregulation in different tumors and the mechanism associated with tumor behavior (formation of the immunosuppressive microenvironment, promotion of tumor angiogenesis, and metastasis). We also summarized and discussed the perspective about the potential application of CXCL5 in tumor therapy targeting the tumor inflammatory microenvironment.

## Introduction

Tumorigenesis involves a continuous, dynamic interaction between cancer cells and the tumor microenvironment (TME). TME is a complex environment around a tumor that includes immune cells, fibroblasts, signaling molecules, and the extracellular matrix (1). The cellular components include heterogeneous subsets of tumor cells, inflammatory cells, immune cells, like monocytes, macrophages, dendritic cells, neutrophils, myelogenous suppressor cells, natural killer cells, T and B cells, as well as mesenchymal stem cells, endothelial cells, cancer-associated fibroblasts. Non-cellular components mainly include cytokines and chemokines. Recent studies have shown that chemokines and their receptors affect tumor progression by regulating the immune responses; they may affect both primary tumors and metastatic tumors. C‐X‐C motif chemokine ligand 5 (CXCL5) is a chemokine that promotes tumor formation by triggering the migration of immune cells to tumors and promotes immmuno-suppressive characteristics of the tumor microenvironment. In addition, CXCL5 can also promote tumor cell metastasis and recruit vascular endothelial cells for angiogenesis. Thus, CXCL5 has been suggested as a novel target for cancer treatment. This review summarizes the advances in research on CXCL5 and the usage of CXCL5 in future cancer treatment.

## Chemokines and CXCL5

Chemokines are a class of pro-inflammatory cytokines composed of 70 ~ 100 amino acids that can recruit and activate chemotactic cells *via* specific seven-transmembrane receptors (2). Chemokine molecular features include four highly conserved cysteine amino acid residues, which form two disulfide bonds, pairing the first with the third cysteine and the second with the fourth cysteine. According to the insertion of other non-conserved amino acids between the first two cysteines (C) close to the N-terminal, chemokines can be divided into four structural branches (**Table 1**) **(** 3, 4): CXC (the first two cysteines separated by one residue amino acid), CC (uninserted amino acid), CX3C (the first two cysteines separated by three residue amino acid), and C (only one cysteine at the N-terminal). Their corresponding receptors are CXCR, CCR, CR, and CX3CR, respectively (5). Additionally, based on the situation whether the N-terminus of the molecule that immediately precedes the first cysteine amino acid residue of CXC chemokine contains a glutamic acid-leucine-arginine motif (‘ELR’ motif; ELR^+^) or not, the CXC family can be further classified. CXC chemokines containing the ‘ELR’ motif (named ELR^+^CXC) are potent promoters of angiogenesis (6, 7).

**Table 1:** chemokine receptors and their ligands.

Chemokines	Other Names	Chromosome	Category	Recepter
CXCL1	GRO-α, MGSA, mouse KC	4q13.3	CXC, ELR	CXCR2
CXCL2	GRO-β, MIP-2α, mouse MIP2	4q13.3	CXC, ELR	CXCR2
CXCL3	GRO-γ, MIP-2β	4q13.3	CXC, ELR	CXCR2
CXCL4	PF4	4q13.3	CXC, non-ELR	CXCR3-B
CXCL4L1	PF4V1	4q13.3	CXC, non-ELR	CXCR3-B
CXCL5	ENA78, SCYB5	4q13.3	CXC, ELR	CXCR2
CXCL6	GCP2	4q13.3	CXC, ELR	CXCR1, CXCR2
CXCL7	NAP-2	4q13.3	CXC, I, ELR	CXCR1, CXCR2
CXCL8	IL-8	4q13.3	CXC, ELR	CXCR1, CXCR2
CXCL9	MIG	4q21.1	CXC, non-ELR	CXCR3
CXCL10	IP-10	4q21.1	CXC, non-ELR	CXCR3
CXCL11	I-TAC	4q21.1	CXC, non-ELR	CXCR3, CXCR7
CXCL12	SDF-1	10q11.21	CXC, non-ELR	CXCR4, CXCR7
CXCL13	BLC, BCA-1	4q21.1	CXC, non-ELR	CXCR5, CXCR3
CXCL14	BRAK	5q31.1	CXC, non-ELR	Unknown
CXCL16	SR-PSOX	17p13.2	CXC	CXCR6
CXCL17	DMC	19q13.2	CXC	Unknown
CCL1	I-309, mouse TCA3	17q11.2	CC	CCR8
CCL2	MCP-1, mouse JE	17q11.2	CC	CCR2
CCL3	MIP-1α, LD78α	17q11.2	CC	CCR1, CCR5
CCL4	MIP-1β	17q12	CC	CCR5
CCL5	RANTES	17q12	CC	CCR1, CCR3, CCR5
CCL7	MCP-3	17q11.2	CC	CCR1, CCR2, CCR3
CCL8	MCP-2	17q11.2	CC	CCR1, CCR2, CCR5
CCL11	Eotaxin	17q11.2	CC	CCR3, CCR5
CCL13	MCP-4	17q11.2	CC	CCR2, CCR3
CCL14	HCC-1	17q12	CC	CCR1, CCR3, CCR5
CCL15	HCC-2 Leukotactin-1	17q12	CC	CCR1, CCR3
CCL16	LEC, HCC-4	17q12	CC	CCR1, CCR2, CCR5, CCR8, H4
CCL17	TARC	16q13	CC	CCR4
CCL18	PARC, DC-CK1	17q12	CC	PITPNM3
CCL19	MIP-3β, ELC	9p13.3	CC	CCR7
CCL20	MIP-3α, LARC	2q36.3	CC	CCR6
CCL21	SLC, 6Ckine	9p13.3	CC	CCR7
CCL22	MDC	16q13	CC	CCR4
CCL23	MPIF-1	17q12	CC	CCR1, FPRL-1
CCL24	Eotaxin-2 MPIF-2	7q11.23	CC	CCR3
CCL25	TECK	19p13.2	CC	CCR9

In recent years, more and more studies have shown that chemokines and chemokine receptors in tumor tissues have important roles in tumorigenesis. Chemokines regulate the immune response of primary and metastatic tumors *via* the recruitment of different immune cell subsets. The pro-tumor or anti-tumor effect depends on which kind of immune cells are recruited (8). Immune cells with potent anti-tumor effects are CD8^+^ T cells (9), polyfunctional TH17 cells (10), and natural killer (NK) cells (11, 12). When these cells are recruited, tumor progression is suppressed. In contrast, the recruitment of tumor-promoting immune cells, such as myeloid-derived suppressor cells (MDSCs) (13), Treg cells (14), TH22 cells (15), and plasmacytoid dendritic cells (pDCs) (16, 17), endows chemokines to promote tumor growth.

Many studies have shown that partial chemokines directly promote tumor cell proliferation and metastasis in an autocrine or paracrine manner. For example, cervical cancer cells treated with exogenous chemokines CXCL3 exhibited enhanced proliferation and migration activities by regulating the expression of the extracellular signal-regulated kinase (ERK) signaling pathway associated genes, including ERK1/2, Bcl-2, and Bax (18). In addition, the angiogenesis effect of chemokine CXCL8 has been confirmed in several cancers, including breast cancer (19), non-small cell lung cancer (NSCLC) (20), melanoma (21), and colorectal cancer (22). Chemokines and their homologous receptors regulate tumor growth in direct and indirect ways: activating signaling pathways to directly regulate the metastasis of tumor cells, working on vascular endothelial cells to indirectly regulate tumor growth, and coordinating the migration and localization of immune cells in tissues.

**Figure 1:**
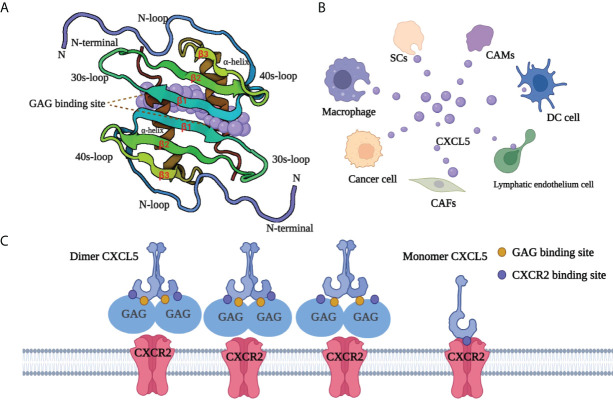
Structure and GAG-binding region of CXCL5. **(A)** The protein dimer comprises of a six-stranded antiparallel β-sheet and a pair of α-helices. The monomer structure consists of an extended N-terminal loop (N-loop) followed by three β- strands and a terminal α-helix. **(B)** CXCL5 can be secreted by various cells, including tumor cells, immune cells, and other non-immune cells. Cancer-associated mesothelial cells (CAMs), cancer-associated fibroblasts (CAFs), dendritic cells (DC cell), Schwann cell (SCs). **(C)** The GAG-binding region and CXCR2-binding region overlap. GAG-bound CXCL5 is unable to activate the receptor CXCR2. CXCL5, C‐X‐C motif chemokine ligand 5; CXCR2, C‐X‐C motif chemokine receptor 2; SCs, Schwann cells; CAMs, cancer-associated mesothelials; DC cell, dendritic cell; CAFs, cancer-associated fibroblasts; GAG, glycosaminoglycan.

CXCL5, also known as neutrophil-activating peptide 78 (ENA-78) or SCYB5 (23), is a member of the CXC chemokine family containing a highly conserved three amino acid motif (ELR^+^) (6). Encoded by the CXCL5 gene (map to chromosome 4q13-q21 and contains 4 exons and 3 introns), CXCL5 has a similar chromosome structure and chromosome location to the IL-8 gene. Clusters of basic chemokines or chemokines containing basic residue can be closely combined with acid mucopolysaccharide glycosaminoglycans (GAGs) containing carboxylate and sulfate moieties by engineering electrostatic/H-bonding complementarity (24). Unlike CXCL1 (25) and CXCL8 (26), the GAG geometry in CXCL5 is novel (**Figure 1**): GAG-binding amino acid residues from a continuous surface layer participate in receptor interactions (27). As a result, when the GAG-binding region and receptor binding region overlap, GAG-bound CXCL5 cannot activate the receptor.

CXCL5 exists both as a monomer and dimer. Meanwhile, each monomer involves the N-loop, 40s loop, β3 strand, and α-helix, which form a continuous surface (28)(**Figure 1**). The precise balance between GAG binding dimer and free soluble monomer regulates CXCL5-mediated receptor response and orderly regulates cells activities. After specifically binding with its recepter CXCR2, CXCL5 can chemotactic a series of biological effects such as the degranulation of neutrophils, lymphocytes and so on, and play an important role in anti-infection and anti-viral immunity. In adipose tissue, CXCL5 acts as a adipokine by activating the JAK2/STAT5 pathway that inhibits insulin signaling and promotes obesity (29). CXCL5 also plays an important biological role in promoting angiogenesis, mediating inflammatory response and participating in connective tissue remodeling. As an inflammatory chemokine, CXCL5 has been associated with inflammatory diseases (30) such as Crohn’s disease, ulcerative colitis, acute appendicitis, and chronic pancreatitis. Additionally, it is believed that CXCL5 can trigger tumor metastasis and promote the formation of an immunosuppressive microenvironment (31–33). Considering its pro-tumor role, CXCL5 has a promising prospect in tumor diagnosis and treatment.

CXCL5 is produced from different sources (**Figure 1**). The role of CXCL5 secreted by immune cells is to participate in the formation of an immunosuppressive microenvironment or promote tumor metastasis. Zhou *et al.* (34) found that CXCL5 released from macrophages in gastric cancer could activate the CXCR2/STAT3 feed-forward under TNF-α (tumor necrosis factor‐α) induced conditions and promote the migration of gastric cancer. Moreover, Fujimura et al. (35) reported that tumor-associated macrophages (TAMs) produce CXCL5. Kuo and colleagues found that tumor-associated dendritic cells (TADCs)-derived CXCL5 participate in some stages of cancer formation and, together with HB-EGF (heparin-binding EGF-like growth factor), can trigger tumor angiogenesis, epithelial-mesenchymal transition (EMT), and increasing proliferation and metastasis of cancer cells (36). Other studies have proved that CXCL5 could also be secreted from tumor cells. For example, Zhao et al. found that in the hepatic metastasis of colonic carcinoma, CXCL5 was highly expressed in tumor cytoplasm and cell membrane, but not tumor mesenchyme (37). In addition, the host cells in TME have also been found to be a non-negligible source of CXCL5. Schwann cells (SCs) in the peripheral nervous system can secrete CXCL5 and increase the motility in tumor cells in the CXCR2/PI3K/AKT/glycogen synthase kinase‐3 (GSK‐3β)/Snail‐Twist signaling pathway, and promote metastasis (38). The high expression of CXCL5 in fibroblasts has also been proven in melanoma (39). Similarly, cancer-associated mesothelial cells (40) with tumor-promoting potential can secrete CXCL5 to promote the metastasis of ovarian cancer cells in the feedback loop (41).

## CXCL5 and cancer

### Overexpression of CXCL5 contributes to tumor progression

According to previous studies, abnormal expression of CXCL5 is found in various malignant tumors. Rajkumar et al. found an increased expression of CXCL5 in gastric cancer tissues compared with adjacent non-cancerous tissues; CXCL5 was also positively correlated with cancer progression and lymphatic metastasis (42). Moreover, Miyazaki and colleagues found that CXCL5 transcription and secretion are significantly upregulated in metastatic cells compared with primary head and neck squamous cell carcinoma and that the interference with its transcription leads to the reduction of the ability of tumor cells to migrate and invade other tissues (43). Also, CXCL5 transcription is significantly upregulated in sporadic endometrioid endometrial adenocarcinoma but not in the normal endometrium (44). Gao *et al.* discovered higher serum CXCL5 concentrations in non-small cell lung cancer patients than in healthy controls (45). High expression of CXCL5 has also been associated with a poor survival rate of pancreatic cancer (46) and with neutrophil infiltration, shortened overall survival, and faster tumor recurrence in hepatocellular carcinoma (47). Other studies have proved that CXCL5 is upregulated in colorectal cancer compared with para-cancerous tissues (37), and its expression is associated with advanced tumor stage, poor prognosis, and liver metastasis (48). Higher levels of CXCL5 in colorectal cancer tissues are also positively correlated with the expression of specific endothelial marker CD31 (49), which provides inspiration for further exploring the role of CXCL5 in tumor angiogenesis (50). In addition, Zheng et al. (51) found that CXCL5-related mRNA and protein were highly expressed in bladder cancer. They found that CXCL5 knockout could significantly inhibit the growth and migration of bladder cancer cells in the medium through Snail, PI3K-AKT, and ERK1/2 signaling pathways.

### CXCL5 is related to different signaling pathways

CXCL5 has been reported to have an indispensable role in promoting tumor progression through different signaling molecules or signaling pathways in different tumors (**Table 2**). For example, overexpression of CXCL5 in colorectal cancer promotes tumor angiogenesis (50), neutrophils recruitment (53), EMT (37), and the formation of immunosuppression microenvironment *via* AKT and ERK pathways (39). Thus, CXCL5 is defined as a promoter of colorectal cancer metastasis and a predictor of poor clinical outcomes in colorectal cancer patients (37). Furthermore, in breast cancer, CXCL5 accelerates neutrophil aggregation and promotes cancer cell colonization and advancement of distant metastasis *via* the ERK/MSK1/Elk-1/snail signaling pathway (54, 55, 72). Studies on lung cancer have also shown that the MAPK and PI3K pathways are related to the tumor-promoting effect of CXCL5 (36, 38, 57). The progression of lung cancer is accelerated by the proliferation, movement, and diffusion of tumor cells. CXCL5 has also been reported to promote tumor progression in different tumors such as gastric cancer (60), liver cancer (62), bladder cancer (65), prostate cancer (68), nasopharyngeal carcinoma, intrahepatic cholangiocarcinoma (58), osteosarcoma (64), cholangiocarcinoma (59), papillary thyroid carcinoma (69), and renal cell carcinoma (71). Notably, CXCL5 also induces the development, metastasis, and drug resistance of bladder cancer. In non-muscle-invasive bladder cancer (NMIBC), resistance to mitomycin C is accompanied by increased expression of tumor-derived CXCL5 (66). Anti-CXCL5 could sensitize tumors to mitomycin C treatment in the nude mouse model. All these data indicated that CXCL5 might activate EMT and the NF-κB (nuclear factor‐κappa B) pathway to promote mitomycin resistance, thus providing a new strategy for discovering new chemical resistance-related markers and overcoming chemical resistance in patients with bladder cancer (66).

**Table 2:** A variety of signaling molecules and pathways are involved in CXCL5 to promote cancer progression.

Cancer type	Function	Signaling molecules	Ref
Colorectal carcinoma	Angiogenesis	AKT, NF-kB	(50)
	EMT and metastasis	ERK/Elk-1/Snail, AKT/GSK3β	(37)
	Immunosuppression	PI3K/AKT	(39)
	Liver metastasis	HSPC111, acetyl-CoA	(52)
	Neutrophils recruitment	Basic leucine zipper transcription factor ATF-like 3	(53)
Breast cancer	Progression	TNFα	(54)
	Progression	ERK/Elk-1/snail	(55)
	Metastasis	S100A14	(56)
Lung cancer	Proliferation	MAPK/ERK1/2, PI3K/AKT	(57)
	Metastasis	PI3K/AKT/GSK-3β/Snail-Twist	(38)
Cholangiocarcinoma	Neutrophils recruitment	PI3K-Akt	(58)
	Cellular metabolism	ERK, β-Catenin, Snai1, MMP2	(59)
Gastric cancer	EMT	ERK, p38	(60)
	Metastasis	STAT3	(34)
Nasopharyngeal carcinoma	Metastasis	ERK/GSK-3β/Snail	(61)
Liver cancer	Migration	The activations of ERK1/2, MAPK and JNK pathways	(62)
	Cell growth	Sox9, PI3K-AKT, ERK1/2	(63)
Osteosarcoma	Migration	NA	(64)
Bladder cancer	Migration	PI3K, AKT, MMP2, MMP9	(65)
	Drug resistance	NF-κB	(66)
Prostate cancer	MDSC recruitment	Hippo-YAP	(67)
	Migration and EMT	ERK, Egr-1, Snail	(68)
Thyroid carcinoma	Metastatic phenotype	AKT, GSK-3β, β-catenin	(69)
	Proliferation	JNK, p38	(70)
Renal carcinoma	Endothelial cell proliferation and recruitment	AKT, NF-κB	(71)

## Mechanism of CXCL5 for promoting tumor progression

### Tumor angiogenesis

Tumor angiogenesis is essential during malignant tumor progression. Chemokines can promote tumor angiogenesis in two ways: (1) by improving the migration and proliferation of endothelial cells by directly acting as tumor angiogenesis medium (73); (2) by indirectly promoting angiogenesis by recruiting leukocytes that produce angiogenic factors in TME (74). The ELR motif first became known for its involvement in the recruitment and activation of neutrophils (75). In 1995, Striter et al. (7) found that the ELR^+^CXC family of chemokines displays disparate angiogenic activity, which can induce endothelial chemotaxis *in vitro* and corneal neovascularization *in vivo*. Later, a significant increase in CXCL5 concentration was observed in the peritoneal fluid of patients with severe endometriosis, a disease characterized by highly vascularized endometrial ectopic tissue proliferation. Researchers speculated that CXCL5 might contribute to the pathogenesis of endometriosis, possibly by promoting neovascularization (76).

**Figure 2:**
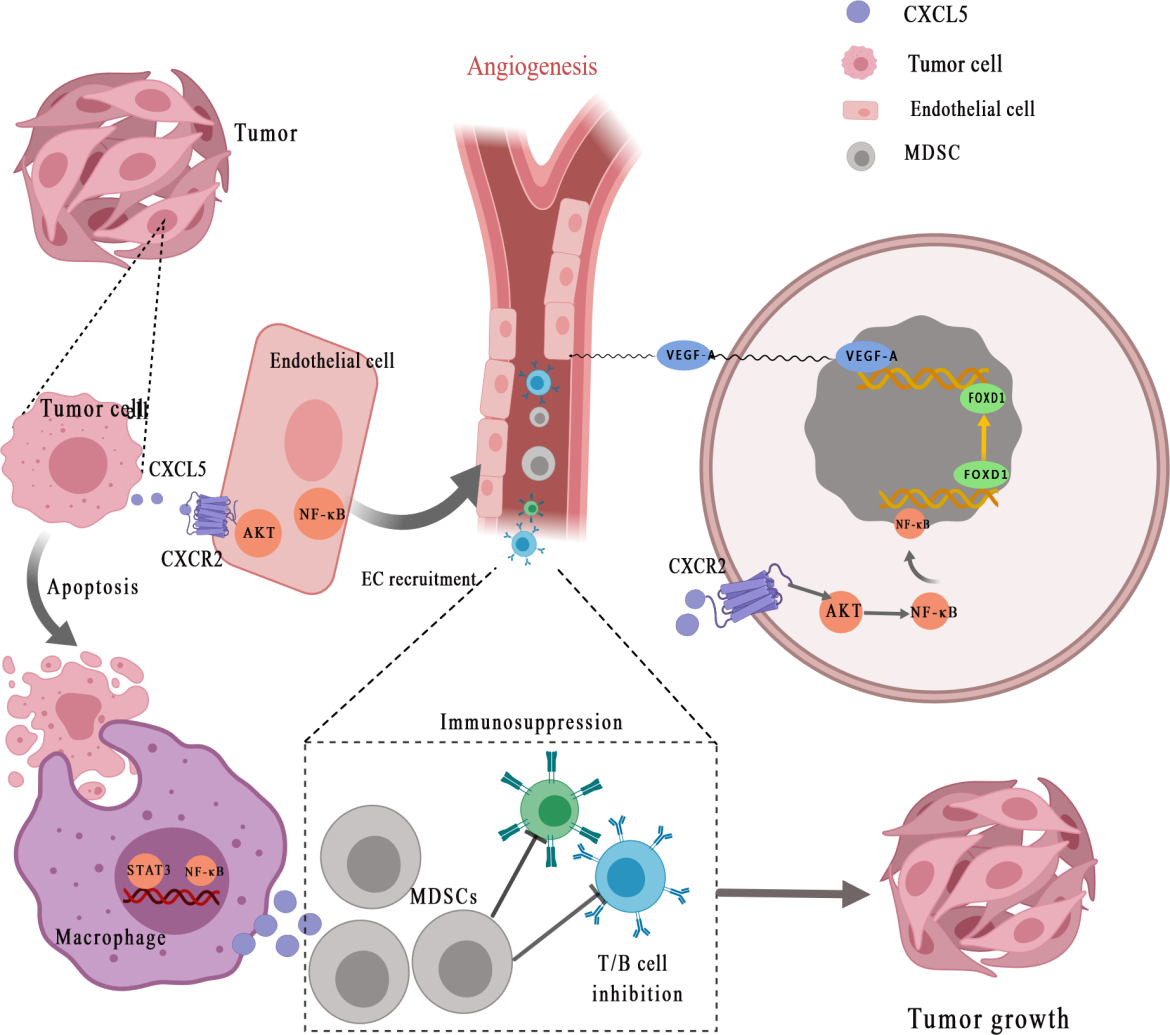
CXCL5 promotes the recruitment of vascular endothelial cells for angiogenesis and helps form the tumor-promoting microenvironment. Tumor cells secret CXCL5 for interaction with its receptor CXCR2, which subsequently activates NF-κB and AKT signaling transduction in endothelial cells. CXCL5 facilitates the recruitment and activation of inflammatory myeloid-derived suppressor cells (MDSCs), eventually leading to persistent inflammation and immunosuppression, thereby stimulating tumor progression. MDSCs, myeloid-derived suppressor cells; EC, endothelial cell; STAT3, signal transducer and activator of transcription 3; NF-κB, nuclear factor‐κappa B; VEGF-A, vascular endothelial growth factor-A; FOXD1, Forkhead Box D1.

A growing number of studies have demonstrated that CXCL5 can promote tumor angiogenesis *via* its highly conserved ELR motif (**Figure 2** **) (** 7). Arenberg et al. (77) first reported that CXCL5, as an angiogenic factor, was elevated in NSCLC tissues and was correlated with vascular density. Angiogenesis factors secreted by cancer cells or stromal cells can directly stimulate angiogenesis. The recent findings suggested that interaction between endothelial cells (ECs) and cancer cells enhances ECs recruitment and promotes cancer progression through the EGFR-NF-κB-CXCL5-CXCR2 pathway in bladder cancer (78). A similar phenomenon was observed in renal cell carcinoma. Furthermore, Guan et al. discovered that androgen receptor signaling promotes renal cell carcinoma progression by increasing endothelial cell proliferation and recruitment by modulating AKT → NF-κB → CXCL5 signaling (71). Additionally, Chen et al. (50) proposed that CXCL5, which is positively correlated with the micro-vessel marker CD31 [expressed by endothelial cells and hematopoietic cells (79)], activates AKT/NF-κB/FOXD1/VEGF-A pathway in a CXCR2-dependent to enhance its tube formation ability. Forkhead box (FOX) proteins, as the upstream transcription regulator of VEGFR-A (vascular endothelial growth factor receptor A), can bind to the VEGF-A promoter to regulate tumor angiogenesis. Specifically, FOX binds to receptor CXCR2, after which CXCL5 promotes the transfer of NF-κB to the nucleus to increase AKT phosphorylation and finally enhances the Forkhead Box D1 (FOXD1) transcriptional activity of VEGF-A promoter, so as to promote tumor angiogenesis. EGR1 (early growth response protein 1) is a transcription factor that has previously been shown to encode a C2H2 type zinc finger protein induced by mitotic stimulation (80), thereby stimulating angiogenesis and improving the survival of tumor cells. CXCL5 can stimulate EGR1 gene transcription to stimulate angiogenesis (81).

### Tumor metastasis

Tumor metastasis is a non-directional and random process. EMT is the initial step in promoting invasive behavior of primary tumors (82, 83). Identified as the down-regulation of epithelial-cadherin (E-cadherin) and the up-regulation of Snails and Vimentin, EMT is characterized by loss of contact between epithelial cells and the separation of junction structures (84). Previous studies have suggested that CXCL5 and its receptors regulate cancer migration and progression through EMT (85). In liver, colon, and lung cancer, PI3K AKT or MAPK (ERK1/2) pathway is involved in CXCL5/CXCR2 activation and cell movement (37, 62). Zhou *et al.* (38) first proposed that CXCL5 is involved in the process of Schwann cells promoting EMT, and in the invasion and metastasis of lung cancer cells *via* the CXCL5/CXCR2/PI3K/AKT/GSK-3β/Snail-Twist pathway. Schwann cells (86), the main glial cells of the peripheral nervous system, have been reported to increase tumor invasiveness in the microenvironment of pancreatic and prostate cancer. Further validation experiments showed that inhibition of CXCL5 or its receptor CXCR2 resulted in decreased expression of snail and twist induced by SC, which in turn reduced the motility of tumor cells (38). In addition, another study found that the overexpressed CXCL5 induces EMT by activating the ERK/Elk-1/Snail pathway and the AKT/GSK3β/β-catenin pathway, thereby enhancing colorectal cancer cell metastasis (37). Furthermore, Cui *et al.* (69) proved that CXCL5 enhances the migration and invasion, and initiates the EMT in papillary thyroid carcinoma cells, a process that can be suppressed by CXCR2 specific short hairpin RNAs (shRNAs). Another study demonstrated that the activated CXCL5-CXCR2 axis contributes to the metastatic phenotype of PTC cells *via* AKT/GSK-3β/β-catenin pathway and accelerates the G1 to S phase transition of papillary thyroid carcinoma cells through JNK and p38 pathways (70). In previous studies, Sex-determining region Y-box 9 (SOX9) was characterized as a CSC marker of hepatocellular carcinoma (HCC), and its expression promoted the growth and invasion of HCC cells in cell cultures (87). Ren *et al.* (63) confirmed that CXCL5 promotes SOX9, which, in turn, promotes the growth and invasion of liver cancer cells through activation of PI3K-Akt and ERK1/2 signaling (63). Gao *et al.* (65) found that CXCL5 activates PI3K/AKT signaling to promote bladder cancer cell EMT and migration through the upregulation of matrix metalloprotein 2 (MMP2) and matrix metalloprotein 9 (MMP9). In addition to PI3K/AKT pathway, CXCL5 also acts on the STAT3 axis. Under TNF-α induced conditions, macrophages release CXCL5, then activate the CXCR2/STAT3 feed-forward loop in a CXCL5-dependent manner (34), thereby promoting the migration of gastric cancer.

**Figure 3:**
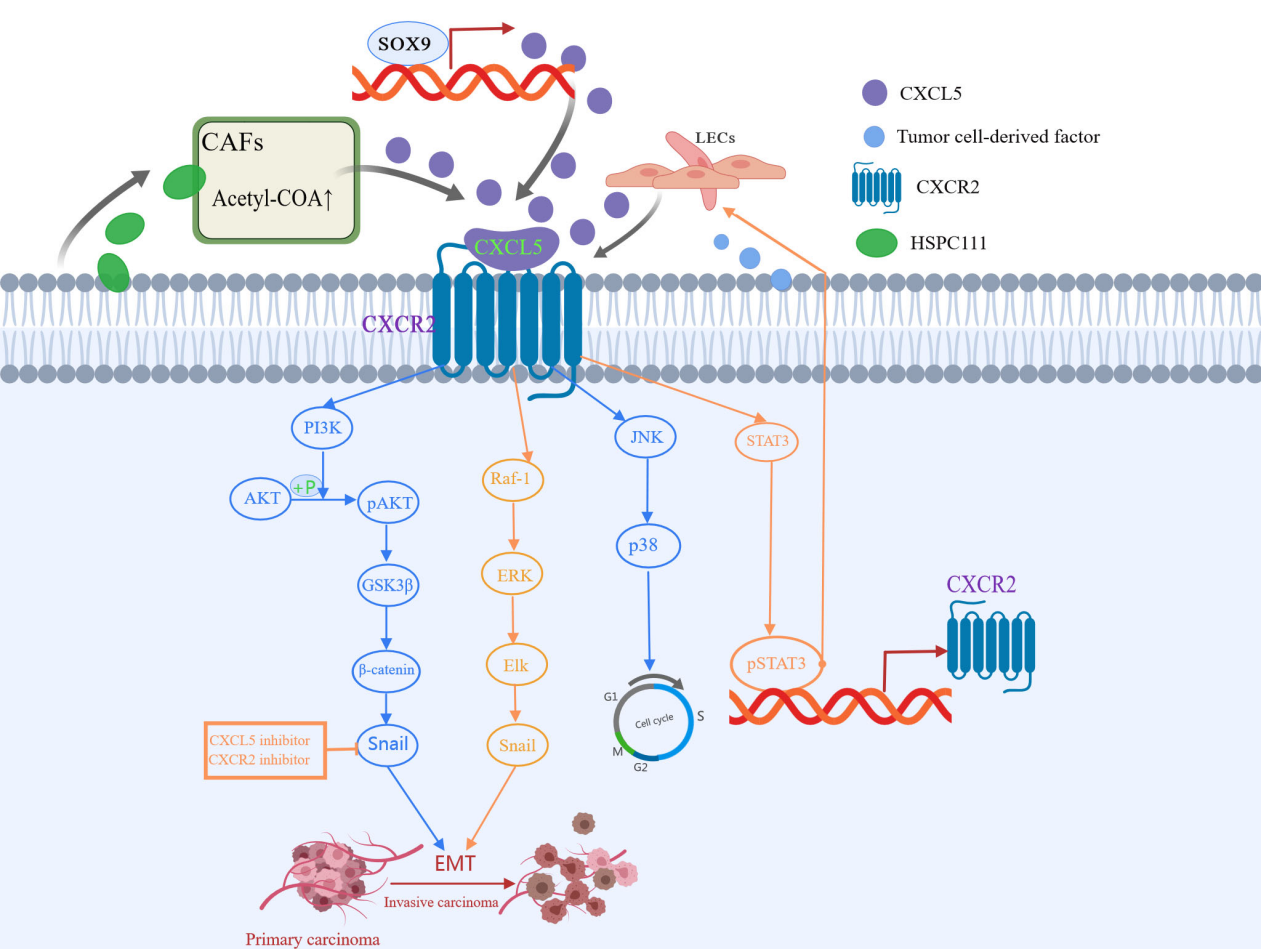
CXCL5 induced in various ways participates in different signaling pathways to promote tumor metastasis. CXCL5-CXCR2 axis is involved in the process of epithelial-mesenchymal transformation (EMT), invasion, and metastasis of cancer cells *via* the PI3K/AKT/GSK-3β/Snail-Twist pathway, ERK/Elk-1/Snail pathway, as well as JNK and p38 pathways.

Metabolic reprogramming of cancer-associated fibroblasts (CAFs) is important for cancer progression (88). Previous studies have elucidated that CAFs crosstalk with cancer cells by secreting various cytokines and chemokines (89). In the latest study, Zhang *et al.* (52) found that HSPC111, an exosome derived from colorectal cancer cells, induces the cancer-promoting factor CXCL5 produced by CAFs to enhance its liver metastasis-promoting effect. Specifically, exosomes HSPC111 upregulates acetyl-CoA’s level in a liver pre-metastatic niche by altering the lipid metabolism of CAFs, which further promotes the secretion of CXCL5. This, in turn, mediates EMT and liver metastasis and enhances the excretion of exosome HSPC111 in a feedforward regulatory loop (52). Moreover, CXCL5 also promotes lymph node metastasis. In the study of the mechanism of lymph node metastasis in HNSCC, Lee *et al.* (90) proved for the first time that CXCL5 and its interaction with receptor CXCR2 is an important link between crosstalk and metastasis between HNSCC and lymphatic endothelial cells. At the same time, they also proposed that the lymphatic endothelium is a novel source of CXCL5 and CXCL5/CXCR2 pathway is a potential therapeutic target for inhibiting lymph node metastasis of head and neck squamous cell carcinoma. All this evidence has proved that as a veritable catalyst for tumor progression, CXCL5 promotes tumor progression in many ways (**Figure 3**), and more ways need to be found.

### Tumor-promoting microenvironment

In cancer development and progression, there is a complex and multidirectional interaction between cancer cells and the tumor inflammatory environment (91). CXCL5, identified as a neutrophil-activated inflammatory peptide homologous to interleukin 8 (92), has been identified as the key mediator for the recruitment of neutrophils to the tumor microenvironment. By secreting immunoreactive molecules such as b2-integrins, onco-statin M, and neutrophil elastase, neutrophils can help form a microenvironment that promotes the migration, invasion, and diffusion of cancer cells (93, 94). In intrahepatic cholangiocarcinoma (95), Zhou *et al.* (58) observed an increase in neutrophil infiltration in the tumor microenvironment with high expression of tumor-derived CXCL5, which confirmed that CXCL5, as a strong chemokine of neutrophils, recruits more neutrophils to establish a tumor-promoting microenvironment and amplifies inflammatory response to accelerate the progression of ICC. CXCL5 participates in and regulates the closed-loop composed of tumor cells, neutrophils, and lymphatic endothelial cells in melanoma (96). Overexpression of tumor-derived CXCL5 recruits many intra and peritumoral neutrophils and promotes lymphangiogenesis, promoting the metastasis of tumor cells from the primary tumor site to local, regional lymph nodes (96).

It is well established that tumor-mediated immune evasion and immunosuppressive microenvironment reduce the clinical efficacy of immunotherapy (97, 98). Myeloid-derived suppressor cells (MDSCs) derived from myeloid progenitor cells inhibit the function of T cells and NK cells, which is considered to have a major role in tumor-mediated immune evasion (99, 100). CXCL5 is a chemoattractant for MDSCs (**Figure 2**) **(** 33). Researchers have found that the clearance of apoptotic cancer cells by macrophages (termed efferocytosis) activates STAT3 and NF-κB [one of the key factors of inflammation-related tumor progression in tumor microenvironment (101)], which stimulates the production of pro-inflammatory cytokine CXCL5 (102). As an inflammatory inducer, CXCL5 facilitates the recruitment and activation of inflammatory myeloid cells and promotes M2 polarization, eventually leading to persistent inflammation in the tumor bone microenvironment and immunosuppression, thereby stimulating tumor progression. Interestingly, the study is also the first to prove that the main contribution of CXCL5 in the tumor microenvironment originated from the host rather than the tumor (102). During the treatment of mesothelioma with oncolytic virotherapy, Tan *et al.* (103) found that CXCL5 derived from tumor cells recruit CXCR2-expressing polymorphonuclear myeloid-derived suppressor cells (PMN-MDSCs) into the tumor microenvironment and suppress dendritic cells to prevent anti-tumor T cell immunity. In addition, through bioinformatics analysis and experimental validation, Wang *et al.* (67) identified that YAP1, the core protein of Hippo pathway (104), is over-activated in prostate tumors. Hyperactivated Hippo-YAP signaling drives the upregulation of CXCL5 in prostate cancer cells through the YAP-TEAD complex and recruits MDSC into TME through CXCL5-CXCR2 signaling. Moreover, abundant MDSCs infiltration exerts an antagonistic effect on T cell proliferation, thus showing potent immunosuppressive activities. At the same time, Wang et al. (67) proved that CXCR2 inhibitors block CXCL5-CXCR2 signaling, resulting in reduced MDSC infiltration and related antitumor effects. In pancreatic ductal adenocarcinoma, the deletion of type I collagen in ASMA^+^ myofibroblasts upregulates the expression of chemokine CXCL5 in cancer cells *via* SOX9 (105). Type I collagen was reported as a significant component of PDAC stroma immune (106). CXCL5 suppress CD8^+^ T cells and recruits MDSC, which are immune cells that inhibit antitumor immune response, thus inhibiting the antitumor immune response.

The expression of programmed death-ligand 1 (PD-L1) has a key regulatory role in tumor immunosuppression of the T cell response. Previous studies have reported that the oncogenic activation of RAS, AKT, or TGF‐β signaling pathways controls the expression of PD-L1 in cancers and has a role in inhibiting tumor immune response (98, 107). In a previous study that investigated the function and regulation of CAFs in the expression of PD-L1 in melanoma and colorectal cancer, researchers described the indispensable role of CXCL5 (39). Firstly, the author identified the abundant expression of CXCL5 in CAFs, and then concluded through further experiments that CXCL5 derived from CAFs promotes the expression of PD-L1 in a concentration‐dependent manner in tumor cells by activating PI3K/Akt signaling, thus mediating the immunosuppressive microenvironment, and this process could be blocked by PI3K inhibitor LY294002 (39).

## Potential clinical applications

### CXCL5 as a predictive biomarker

Cancer is characterized by a high incidence rate, high mortality rate, and heavy economic burden, thus representing the major public health problem in the world. Therefore, searching for new potential prognostic biomarkers has always been one of the main focuses in oncology. Considering the important functions of CXCL5 in tumor differentiation, advanced tumor stages, local invasion, neutrophil infiltration, and metastasis, CXCL5 has been considered a potential biomarker for prognosis and a novel preventive and therapeutic target for cancer.

In a previous study, CXCL5 expression was identified as a predictive biomarker associated with the response and prognosis of immunotherapy in patients with non-small cell lung cancer (108). In addition, the expression of CXCL5 has been associated with poor prognosis in many cancers (109), such as bone-metastatic prostate cancer (68), bladder cancer (110), hepatocellular carcinoma (111), and pancreatic cancer (112). In order to explore the prognostic value of abnormal expression of CXCL5 in cancer patients, Wu et al. (109) conducted a comprehensive meta-analysis and concluded that the high expression level of CXCL5 was significantly correlated with the poor prognosis of cancer patients.

Furthermore, studies suggested that CXCL5 can predict the efficacy and prognosis and predict the adverse reactions. Fujimura et al. (35) detected the concentration of CXCL5 before and after nivolumab treatment. Compared with patients without immune-related adverse events (irAEs), the absolute serum levels of CXCL5 were significantly increased in patients with irAEs. Therefore, CXCL5 has been recommended as a prognostic biomarker of irAEs in patients with advanced melanoma treated with nivolumab.

As a small peptide, CXCL5 has been proved to be secreted from a variety of sources and exists in body fluids. Its abnormal expression in the tumor microenvironment allows us to detect it through blood detection of tumor tissue samples. Therefore, CXCL5 can be used as a convenient and fast biomarker to predict the curative effect or prognosis of cancer patients together with other tumor markers.

### As a possible therapeutic target

Owing to the critical role in promoting angiogenesis, tumor metastasis, and the formation of the immunosuppressive microenvironment, targeting CXCL5 brings more possibilities for cancer therapy. Since CXCL5 promotes tumor angiogenesis by controlling the expression and transcriptional activity of FOXD1, suppression of angiogenesis by inhibiting the CXCL5/CXCR2 axis may be a promising treatment for colorectal patients (50). Kuo *et al.* suggested that TADCs-derived CXCL5 and HB-EGF synergized with the Akt and ERK pathways to promote lung cancer metastasis. Further studies found that neutralizing CXCL5 with its antibody significantly reduces the incidence of cancer progression and enhances the efficacy of TKI (36). Another study found that LY294002 (PI3K inhibitor) blocks the phosphorylation of Akt and GSK-3β in the CXCL5/CXCR2 axis and reduces the spreading and metastasis ability of lung cancer (38). In the study of pancreatic cancer, blocking chemokine signal transduction proved to reverse the anti-tumor immune characteristics induced by type 1 collagen deficiency and slow down the progression of pancreatic cancer (105). Furthermore, the intervention of the TGF-β/Axl/CXCL5 signaling has been suggested as a therapeutic strategy for treating HCC progression in TGF-β-positive patients (113).

The drug sensitivity may vary from patient to patient. CXCL5 can promote mitomycin resistance by activating EMT and NF-κB pathways. Therefore, the identification of CXCL5 as a new marker of chemical resistance may provide a new strategy to overcome drug resistance (66). The interaction between TME and tumor cells has brought a new revolution in anti‐cancer immunotherapy. In exploring improving the homing of NK cells, NK cells transduced with the virus vector encoding CXCR2 show better motility to renal carcinoma expressing the homologous ligand CXCL5. Recent study revealed that blocking the CXCL5/CXCR2 axis with CXCR2 inhibitors prevents the recruitment of inhibitory MDSCs and improves the utility of immunotherapy by enhancing the efficiency of anti‐PD‐1 (114). In addition, inhibition of the CXCL5/CXCR2 axis could reduce the recruitment of granulocytes to the primary tumor area, and inhibit the establishment of early metastatic niches (115). Besides, CXCL5 inhibitors demonstrated a good tolerance in most of the patients clinically, and relevant clinical trials are currently underway. CXCL5-GAG interaction prevent the rapid diffusion of CXCL5 in the circulation, and is the key to form a local concentration gradient. GAG-bound and soluble chemokine gradients mediate neutrophil recruitment in the vasculature and extracellular matrix, which could be exploited for designing inhibitors that disrupt CXCL5-GAG interactions and neutrophil homing to the target tissue (116).

## Conclusion

Recently, CXCL5 has been strongly associated with tumorigenesis and tumor progression through different pathways. CXCL5 has also been associated with the prognosis and diagnosis of cancer therapy. Blocking of CXCL5-associated pathways is an effective method to resist tumor cell growth and improve therapeutic sensitivity. Yet, since current studies are still in the early stage, more factors related to CXCL5 need to be investigated. Despite these challenges, the potential of CXCL5 in cancer screening and personalized anti-cancer treatment is worthy of further exploration.

## Data availability statement

The original contributions presented in the study are included in the article/supplementary material. Further inquiries can be directed to the corresponding author.

## Author contributions

HW conceived the work. JD, RJ, EM, and HW co-wrote the paper. JD prepared the figures. All of the authors discussed the results and commented on the manuscript. All of the authors have read and approved the final manuscript.

## Funding

This work was supported by a grant from the National Natural Science Foundation of China (No. 81301898) and Beijing xisike clinical oncology research foundation (sy2018-249).

## Conflict of interest

The authors declare that the research was conducted in the absence of any commercial or financial relationships that could be construed as a potential conflict of interest.

## Publisher’s note

All claims expressed in this article are solely those of the authors and do not necessarily represent those of their affiliated organizations, or those of the publisher, the editors and the reviewers. Any product that may be evaluated in this article, or claim that may be made by its manufacturer, is not guaranteed or endorsed by the publisher.

## Glossary

**Table:**

C	cysteine
CAF	cancer-associated fibroblast
CAM	cancer-associated mesothelial
CXCL1	C‐X‐C motif chemokine ligand 1
CXCL3	C‐X‐C motif chemokine ligand 3
CXCL5	C‐X‐C motif chemokine ligand 5
CXCL8	C‐X‐C motif chemokine ligand 8
CXCR2	C‐X‐C motif chemokinereceptor 2
DC cell	dendritic cell
EC	endothelial cell
E-cadherin	epithelial-cadherin
EGR1	early growth response protein 1
ELR	Glu Leu Arg sequence
EMT	epithelial mesenchymal transformation
ENA-78	neutrophil activating peptide 78
ERK	extracellular signal-regulated kinase
FOXD1	Forkhead Box D1
GAG	glycosaminoglycan
GSK‐3b	Glycogen synthase kinase‐3
HB-EGF	heparin-binding EGF-like growth factor
HCC	hepatocellular carcinoma
HNSCC	head and neck squamous cell carcinoma
ICC	intrahepatic cholangiocarcinoma
irAEs	immune-related adverse events
LECs	lymphatic endothelial cells
MDSC	myeloid-derived suppressor cell
MMP2	matrix metalloprotein 2
MMP9	matrix metalloprotein 9
NF-kB	nuclear factor‐kappa B
NK cell	natural killer cell
NMIBC	non-muscle invasive bladder cancer
NSCLC	non-small cell lung cancer
pDC	plasmacytoid dendritic cell
PD-L1	programmed death-ligand 1
PMN-MDSC	polymorphonuclear myeloid-derived suppressor cell
SC	Schwann cell
shRNA	short hairpin RNA
SOX9	Sex-determining region Y-box 9
STAT3	signal transducer and activator of transcription 3
TADC	tumor associated dendritic cell
TAM	tumor-associated macrophage
TME	tumor microenvironment
TNF‐a	tumor necrosis factor‐a
VEGF-A	vascular endothelial growth factor A
VEGFR-A	vascular endothelial growth factor receptor A.
